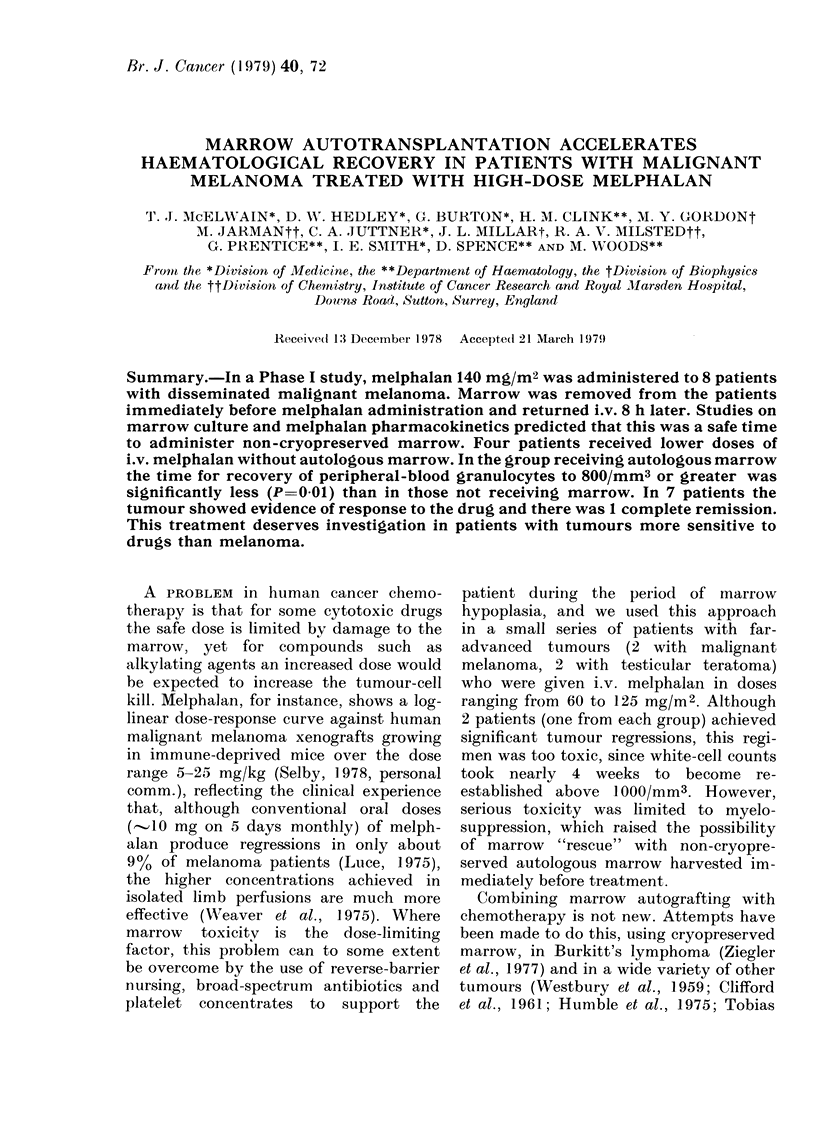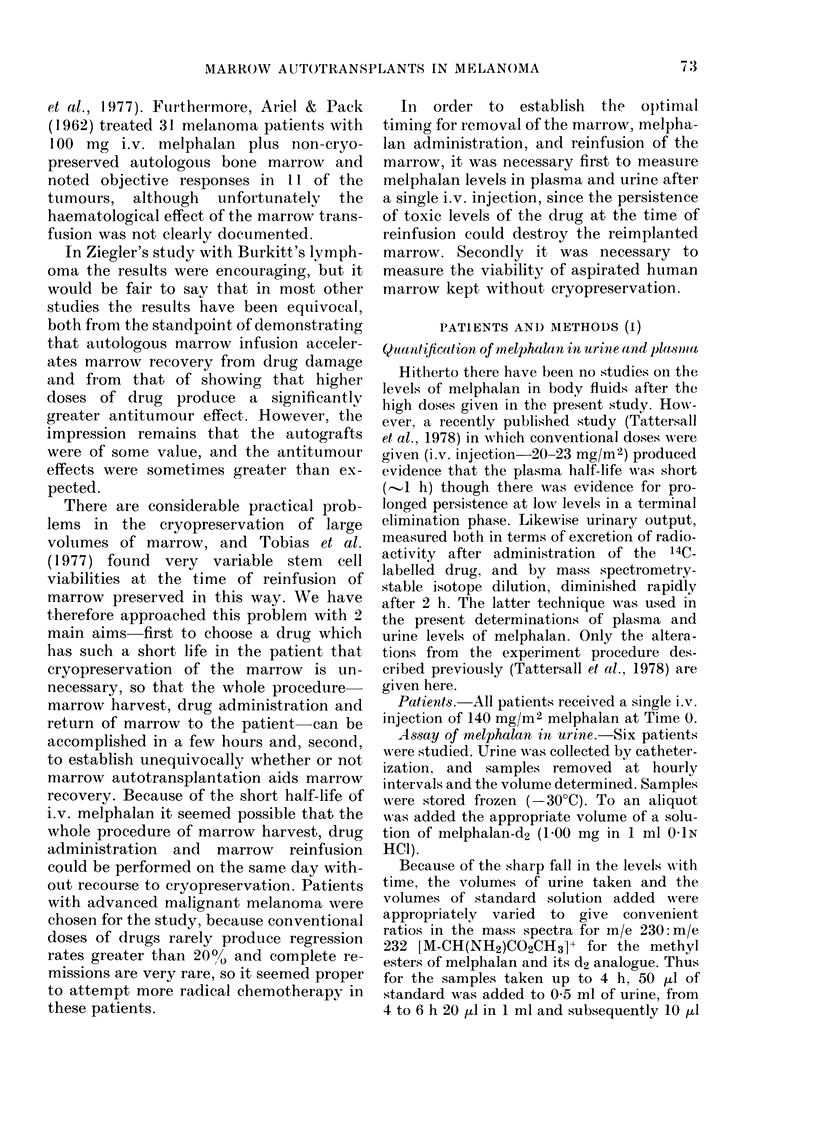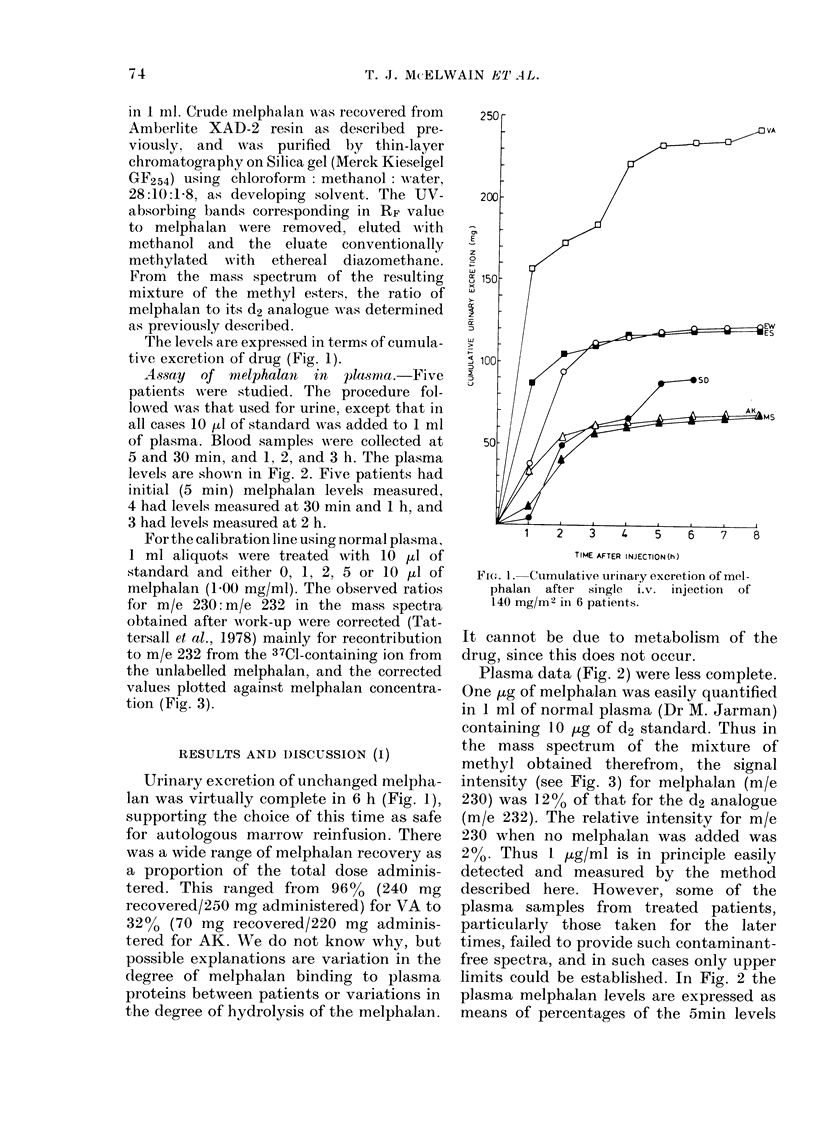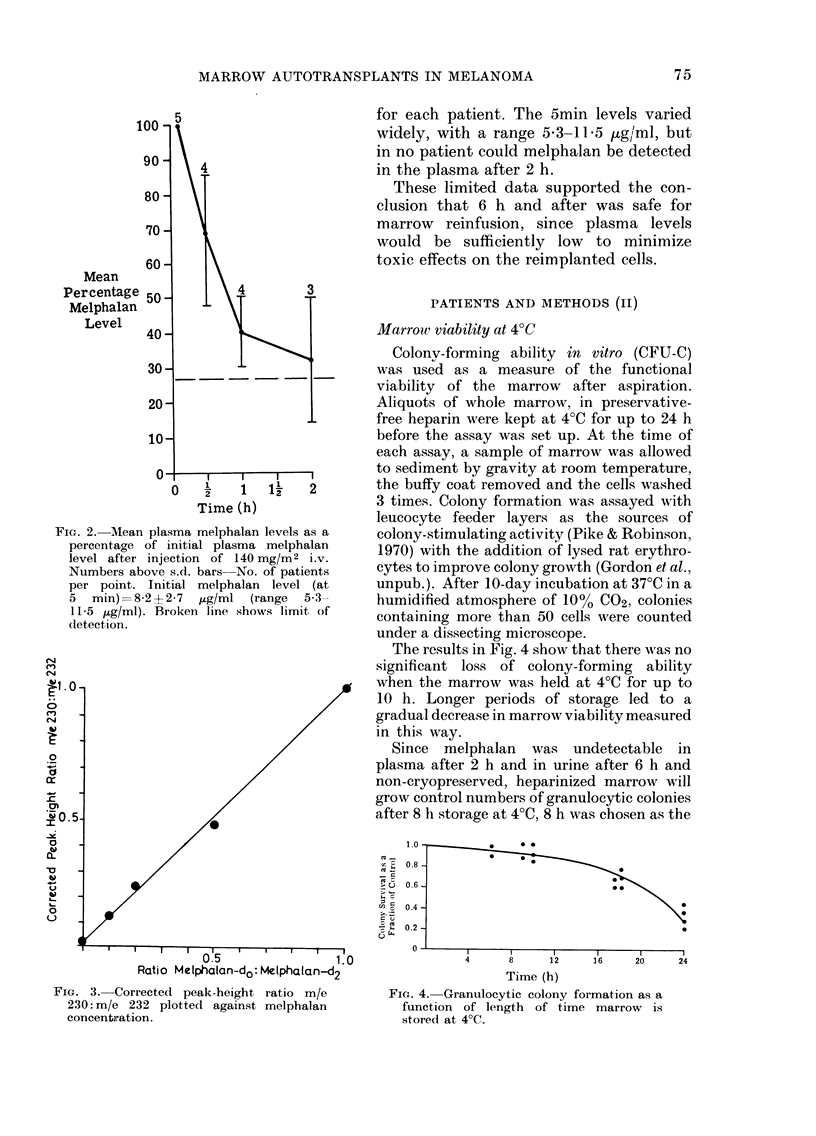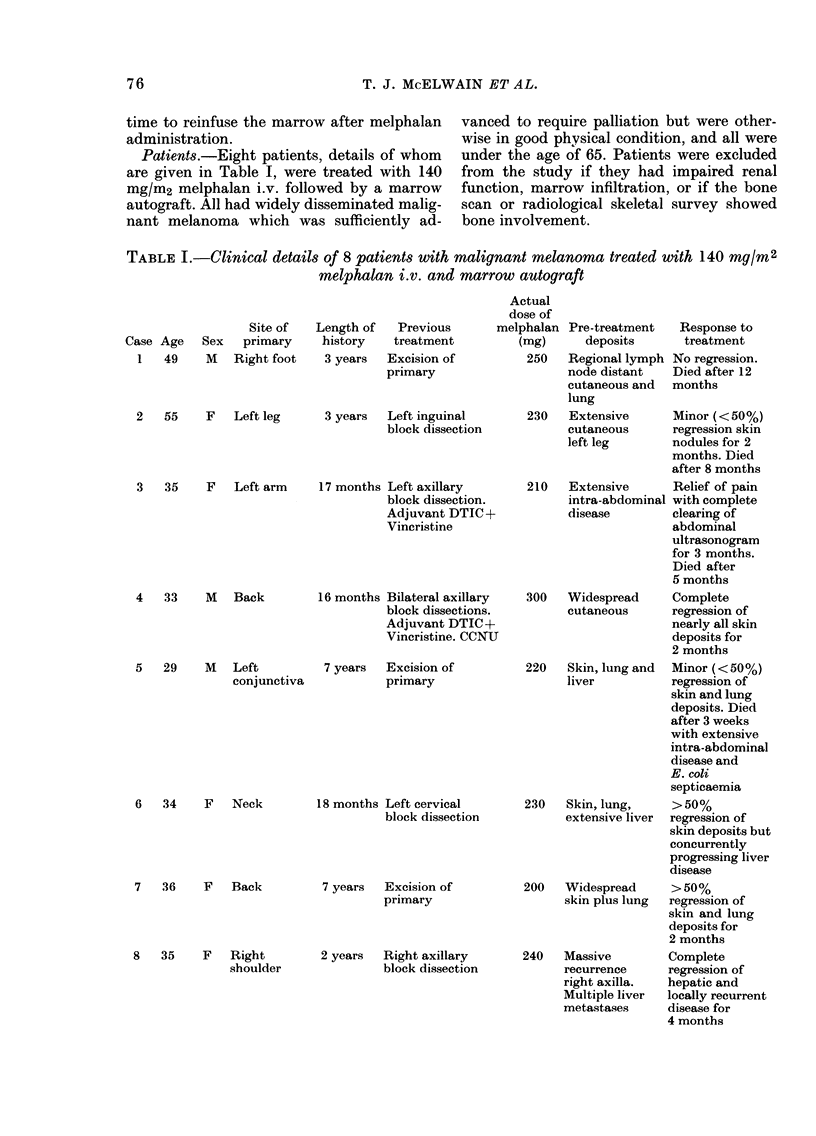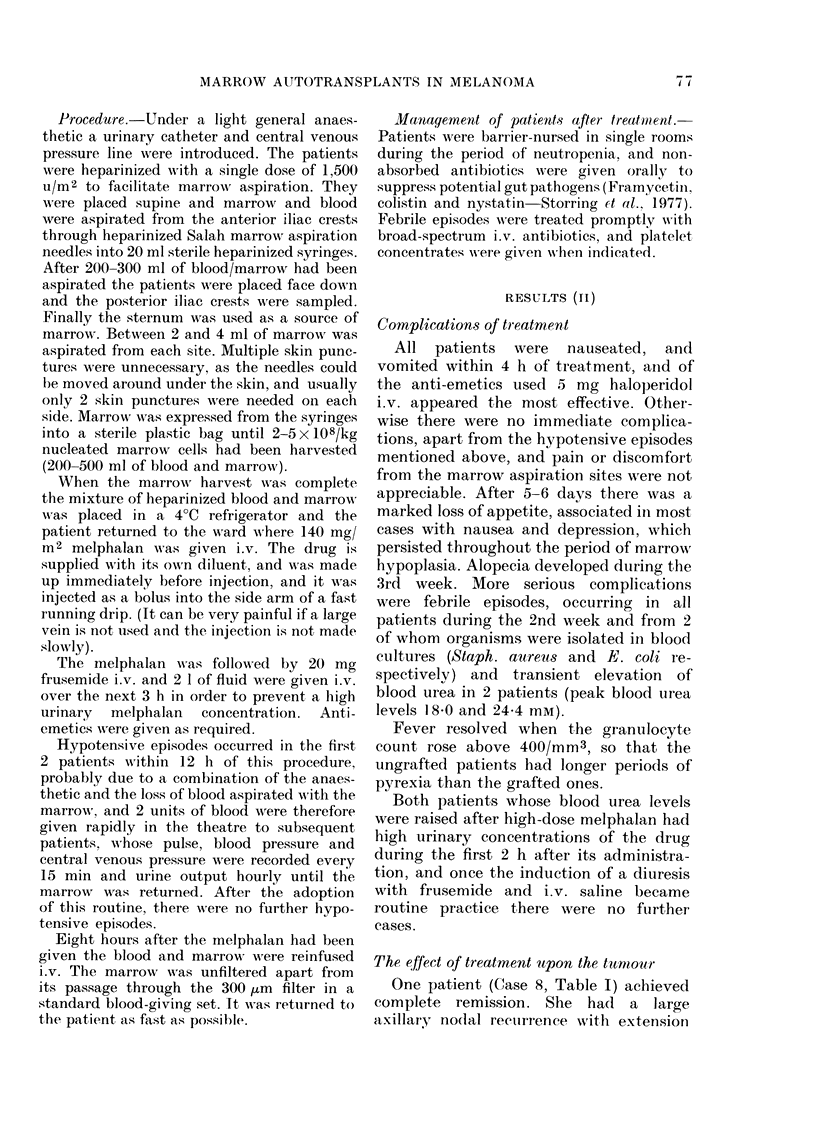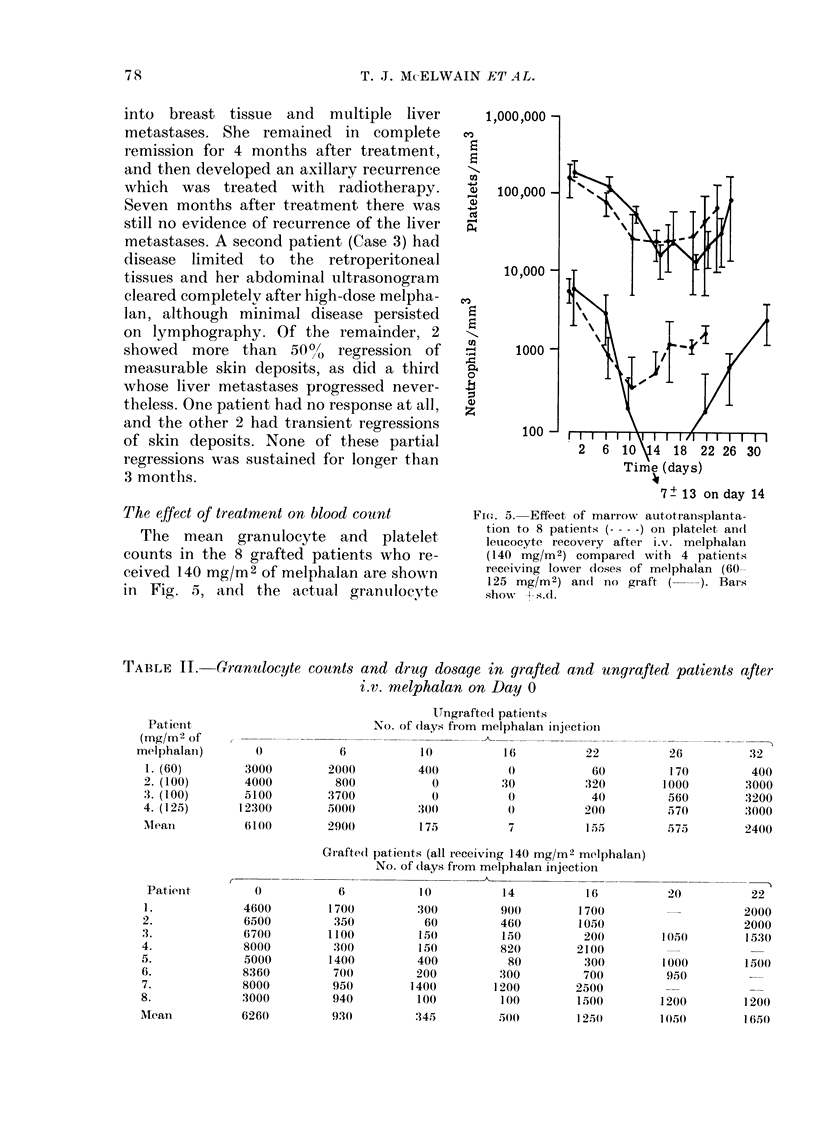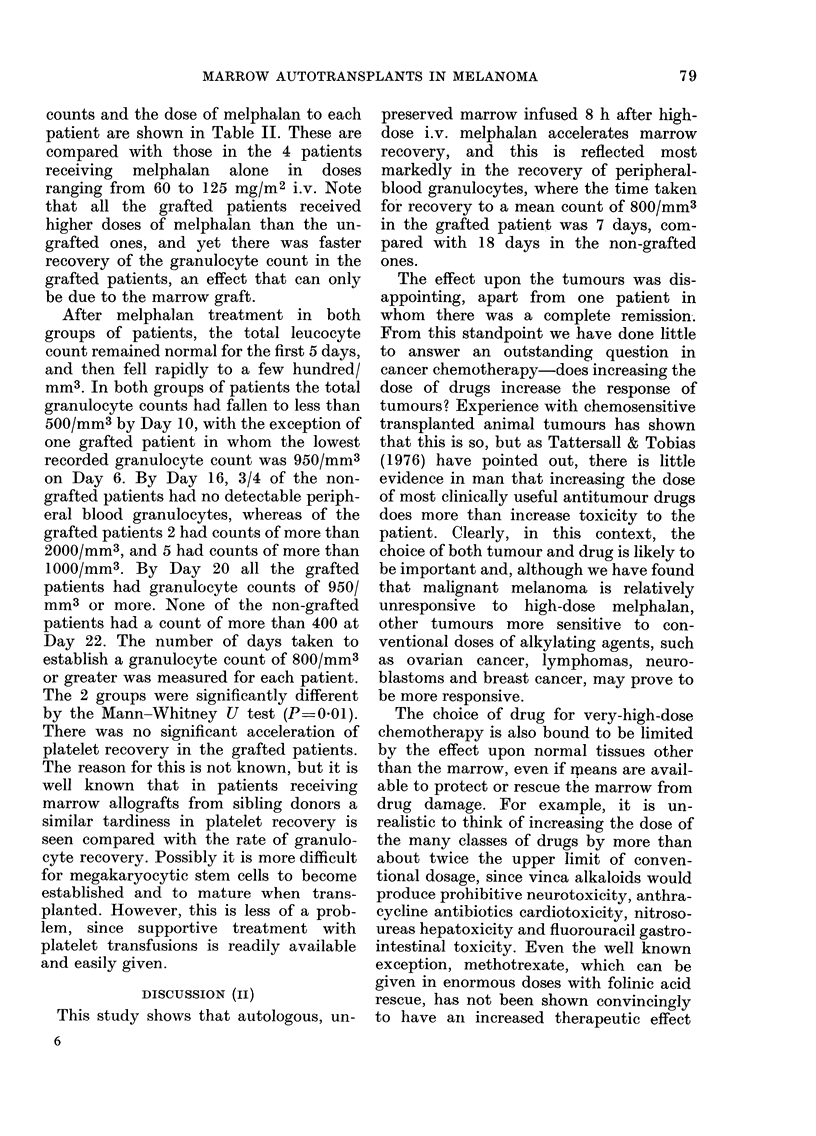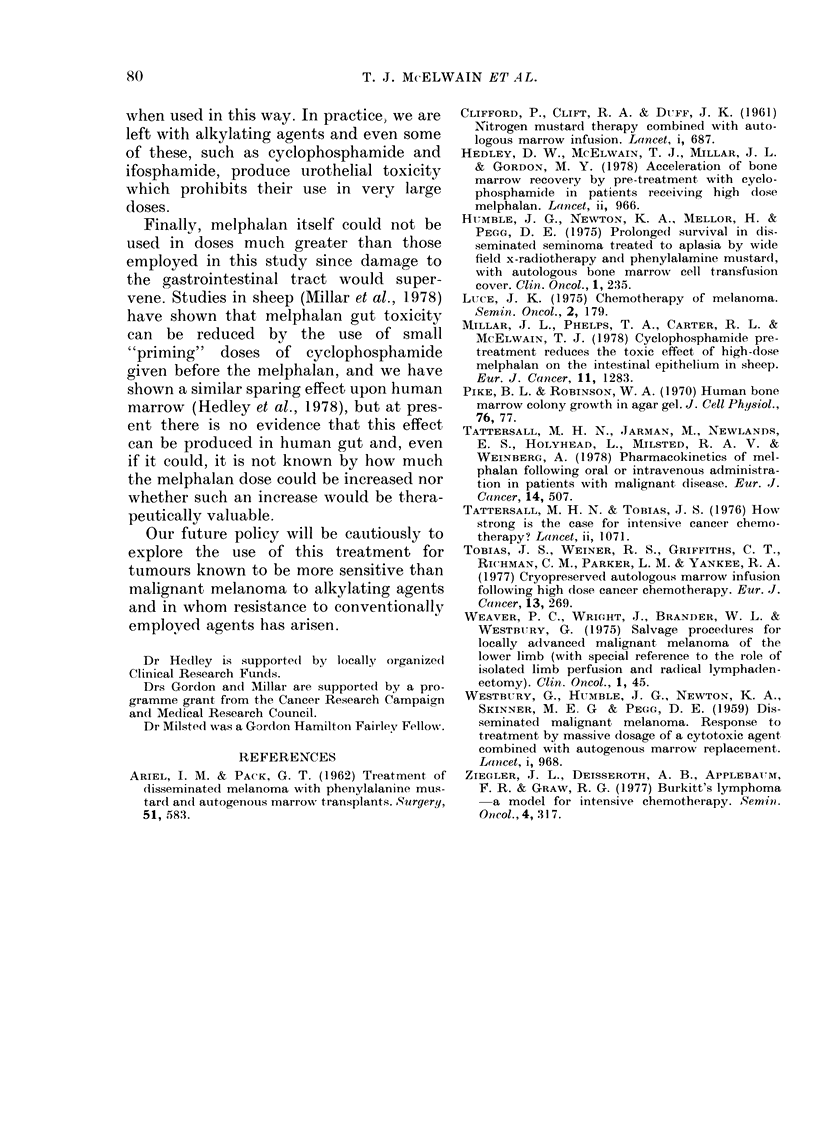# Marrow autotransplantation accelerates haematological recovery in patients with malignant melanoma treated with high-dose melphalan.

**DOI:** 10.1038/bjc.1979.142

**Published:** 1979-07

**Authors:** T. J. McElwain, D. W. Hedley, G. Burton, H. M. Clink, M. Y. Gordon, M. Jarman, C. A. Juttner, J. L. Millar, R. A. Milsted, G. Prentice, I. E. Smith, D. Spence, M. Woods

## Abstract

In a Phase I study, melphalan 140 mg/m2 was administered to 8 patients with disseminated malignant melanoma. Marrow was removed from the patients immediately before melphalan administration and returned i.v. 8 h later. Studies on marrow culture and melphalan pharmacokinetics predicted that this was a safe time to administer non-cryopreserved marrow. Four patients received lower doses of i.v. melphalan without autologous marrow. In the group receiving autologous marrow the time for recovery of peripheral-blood granulocytes to 800/mm2 or greater was significantly less (P = 0.01) than in those not receiving marrow. In 7 patients the tumour showed evidence of response to the drug and there was 1 complete remission. This treatment deserves investigation in patients with tumours more sensitive to drugs than melanoma.


*Br. J. Cancer* (1979) **40**, 72

### MARROW AUTOTRANSPLANTATION ACCELERATES HAEMATOLOGICAL RECOVERY IN PATIENTS WITH MALIGNANT MELANOMA TREATED WITH HIGH-DOSE MELPHALAN

T. J. McELWAIN*, D. W. HEDLEY*, G. BURTON*, H. M. CLINK**, M. Y. GORDON†

M. JARMAN††, C. A. JUTTNER*, J. L. MILLAR†, R. A. V. MILSTED††,

G. PRENTICE**, I. E. SMITH*, D. SPENCE** AND M. WOODS**

*From the *Division of Medicine, the **Department of Haematology, the †Division of Biophysics and the ††Division of Chemistry, Institute of Cancer Research and Royal Marsden Hospital, Downs Road, Sutton, Surrey, England*

Received 13 December 1978 Accepted 21 March 1979

**Summary.—In a Phase I study, melphalan 140 mg/m$^2$ was administered to 8 patients with disseminated malignant melanoma. Marrow was removed from the patients immediately before melphalan administration and returned i.v. 8 h later. Studies on marrow culture and melphalan pharmacokinetics predicted that this was a safe time to administer non-cryopreserved marrow. Four patients received lower doses of i.v. melphalan without autologous marrow. In the group receiving autologous marrow the time for recovery of peripheral-blood granulocytes to 800/mm$^3$ or greater was significantly less ($P = 0{\cdot}01$) than in those not receiving marrow. In 7 patients the tumour showed evidence of response to the drug and there was 1 complete remission. This treatment deserves investigation in patients with tumours more sensitive to drugs than melanoma.**

A PROBLEM in human cancer chemotherapy is that for some cytotoxic drugs the safe dose is limited by damage to the marrow, yet for compounds such as alkylating agents an increased dose would be expected to increase the tumour-cell kill. Melphalan, for instance, shows a log-linear dose-response curve against human malignant melanoma xenografts growing in immune-deprived mice over the dose range 5–25 mg/kg (Selby, 1978, personal comm.), reflecting the clinical experience that, although conventional oral doses (~10 mg on 5 days monthly) of melphalan produce regressions in only about 9% of melanoma patients (Luce, 1975), the higher concentrations achieved in isolated limb perfusions are much more effective (Weaver *et al.*, 1975). Where marrow toxicity is the dose-limiting factor, this problem can to some extent be overcome by the use of reverse-barrier nursing, broad-spectrum antibiotics and platelet concentrates to support the patient during the period of marrow hypoplasia, and we used this approach in a small series of patients with far-advanced tumours (2 with malignant melanoma, 2 with testicular teratoma) who were given i.v. melphalan in doses ranging from 60 to 125 mg/m$^2$. Although 2 patients (one from each group) achieved significant tumour regressions, this regimen was too toxic, since white-cell counts took nearly 4 weeks to become re-established above 1000/mm$^3$. However, serious toxicity was limited to myelosuppression, which raised the possibility of marrow "rescue" with non-cryopreserved autologous marrow harvested immediately before treatment.

Combining marrow autografting with chemotherapy is not new. Attempts have been made to do this, using cryopreserved marrow, in Burkitt's lymphoma (Ziegler *et al.*, 1977) and in a wide variety of other tumours (Westbury *et al.*, 1959; Clifford *et al.*, 1961; Humble *et al.*, 1975; Tobias

*et al.*, 1977). Furthermore, Ariel & Pack (1962) treated 31 melanoma patients with 100 mg i.v. melphalan plus non-cryopreserved autologous bone marrow and noted objective responses in 11 of the tumours, although unfortunately the haematological effect of the marrow transfusion was not clearly documented.

In Ziegler's study with Burkitt's lymphoma the results were encouraging, but it would be fair to say that in most other studies the results have been equivocal, both from the standpoint of demonstrating that autologous marrow infusion accelerates marrow recovery from drug damage and from that of showing that higher doses of drug produce a significantly greater antitumour effect. However, the impression remains that the autografts were of some value, and the antitumour effects were sometimes greater than expected.

There are considerable practical problems in the cryopreservation of large volumes of marrow, and Tobias *et al.* (1977) found very variable stem cell viabilities at the time of reinfusion of marrow preserved in this way. We have therefore approached this problem with 2 main aims—first to choose a drug which has such a short life in the patient that cryopreservation of the marrow is unnecessary, so that the whole procedure—marrow harvest, drug administration and return of marrow to the patient—can be accomplished in a few hours and, second, to establish unequivocally whether or not marrow autotransplantation aids marrow recovery. Because of the short half-life of i.v. melphalan it seemed possible that the whole procedure of marrow harvest, drug administration and marrow reinfusion could be performed on the same day without recourse to cryopreservation. Patients with advanced malignant melanoma were chosen for the study, because conventional doses of drugs rarely produce regression rates greater than 20% and complete remissions are very rare, so it seemed proper to attempt more radical chemotherapy in these patients.

In order to establish the optimal timing for removal of the marrow, melphalan administration, and reinfusion of the marrow, it was necessary first to measure melphalan levels in plasma and urine after a single i.v. injection, since the persistence of toxic levels of the drug at the time of reinfusion could destroy the reimplanted marrow. Secondly it was necessary to measure the viability of aspirated human marrow kept without cryopreservation.

### PATIENTS AND METHODS (I)

### *Quantification of melphalan in urine and plasma*

Hitherto there have been no studies on the levels of melphalan in body fluids after the high doses given in the present study. However, a recently published study (Tattersall *et al.*, 1978) in which conventional doses were given (i.v. injection—20–23 mg/m$^2$) produced evidence that the plasma half-life was short ($\sim$1 h) though there was evidence for prolonged persistence at low levels in a terminal elimination phase. Likewise urinary output, measured both in terms of excretion of radioactivity after administration of the $^{14}$C-labelled drug, and by mass spectrometry-stable isotope dilution, diminished rapidly after 2 h. The latter technique was used in the present determinations of plasma and urine levels of melphalan. Only the alterations from the experiment procedure described previously (Tattersall *et al.*, 1978) are given here.

*Patients.*—All patients received a single i.v. injection of 140 mg/m$^2$ melphalan at Time 0.

*Assay of melphalan in urine.*—Six patients were studied. Urine was collected by catheterization, and samples removed at hourly intervals and the volume determined. Samples were stored frozen (−30°C). To an aliquot was added the appropriate volume of a solution of melphalan-d$_2$ (1·00 mg in 1 ml 0·1N HCl).

Because of the sharp fall in the levels with time, the volumes of urine taken and the volumes of standard solution added were appropriately varied to give convenient ratios in the mass spectra for m/e 230: m/e 232 [M-CH(NH$_2$)CO$_2$CH$_3$]$^+$ for the methyl esters of melphalan and its d$_2$ analogue. Thus for the samples taken up to 4 h, 50 $\mu$l of standard was added to 0·5 ml of urine, from 4 to 6 h 20 $\mu$l in 1 ml and subsequently 10 $\mu$l in 1 ml. Crude melphalan was recovered from Amberlite XAD-2 resin as described previously, and was purified by thin-layer chromatography on Silica gel (Merck Kieselgel $GF_{254}$) using chloroform : methanol : water, 28:10:1·8, as developing solvent. The UV-absorbing bands corresponding in $R_F$ value to melphalan were removed, eluted with methanol and the eluate conventionally methylated with ethereal diazomethane. From the mass spectrum of the resulting mixture of the methyl esters, the ratio of melphalan to its $d_2$ analogue was determined as previously described.

The levels are expressed in terms of cumulative excretion of drug (Fig. 1).

*Assay of melphalan in plasma.*—Five patients were studied. The procedure followed was that used for urine, except that in all cases 10 $\mu$l of standard was added to 1 ml of plasma. Blood samples were collected at 5 and 30 min, and 1, 2, and 3 h. The plasma levels are shown in Fig. 2. Five patients had initial (5 min) melphalan levels measured, 4 had levels measured at 30 min and 1 h, and 3 had levels measured at 2 h.

For the calibration line using normal plasma, 1 ml aliquots were treated with 10 $\mu$l of standard and either 0, 1, 2, 5 or 10 $\mu$l of melphalan (1·00 mg/ml). The observed ratios for m/e 230:m/e 232 in the mass spectra obtained after work-up were corrected (Tattersall *et al.*, 1978) mainly for recontribution to m/e 232 from the $^{37}$Cl-containing ion from the unlabelled melphalan, and the corrected values plotted against melphalan concentration (Fig. 3).

### RESULTS AND DISCUSSION (I)

Urinary excretion of unchanged melphalan was virtually complete in 6 h (Fig. 1), supporting the choice of this time as safe for autologous marrow reinfusion. There was a wide range of melphalan recovery as a proportion of the total dose administered. This ranged from 96% (240 mg recovered/250 mg administered) for VA to 32% (70 mg recovered/220 mg administered for AK. We do not know why, but possible explanations are variation in the degree of melphalan binding to plasma proteins between patients or variations in the degree of hydrolysis of the melphalan.

FIG. 1.—Cumulative urinary excretion of melphalan after single i.v. injection of 140 mg/m$^2$ in 6 patients.

It cannot be due to metabolism of the drug, since this does not occur.

Plasma data (Fig. 2) were less complete. One $\mu$g of melphalan was easily quantified in 1 ml of normal plasma (Dr M. Jarman) containing 10 $\mu$g of $d_2$ standard. Thus in the mass spectrum of the mixture of methyl obtained therefrom, the signal intensity (see Fig. 3) for melphalan (m/e 230) was 12% of that for the $d_2$ analogue (m/e 232). The relative intensity for m/e 230 when no melphalan was added was 2%. Thus 1 $\mu$g/ml is in principle easily detected and measured by the method described here. However, some of the plasma samples from treated patients, particularly those taken for the later times, failed to provide such contaminant-free spectra, and in such cases only upper limits could be established. In Fig. 2 the plasma melphalan levels are expressed as means of percentages of the 5min levels

FIG. 2.—Mean plasma melphalan levels as a percentage of initial plasma melphalan level after injection of 140 mg/m² i.v. Numbers above s.d. bars—No. of patients per point. Initial melphalan level (at 5 min) = 8·2 ± 2·7 μg/ml (range 5·3–11·5 μg/ml). Broken line shows limit of detection.

FIG. 3.—Corrected peak-height ratio m/e 230: m/e 232 plotted against melphalan concentration.

for each patient. The 5min levels varied widely, with a range 5·3–11·5 μg/ml, but in no patient could melphalan be detected in the plasma after 2 h.

These limited data supported the conclusion that 6 h and after was safe for marrow reinfusion, since plasma levels would be sufficiently low to minimize toxic effects on the reimplanted cells.

### PATIENTS AND METHODS (II)

*Marrow viability at 4°C*

Colony-forming ability *in vitro* (CFU-C) was used as a measure of the functional viability of the marrow after aspiration. Aliquots of whole marrow, in preservative-free heparin were kept at 4°C for up to 24 h before the assay was set up. At the time of each assay, a sample of marrow was allowed to sediment by gravity at room temperature, the buffy coat removed and the cells washed 3 times. Colony formation was assayed with leucocyte feeder layers as the sources of colony-stimulating activity (Pike & Robinson, 1970) with the addition of lysed rat erythrocytes to improve colony growth (Gordon *et al.*, unpub.). After 10-day incubation at 37°C in a humidified atmosphere of 10% $CO_2$, colonies containing more than 50 cells were counted under a dissecting microscope.

The results in Fig. 4 show that there was no significant loss of colony-forming ability when the marrow was held at 4°C for up to 10 h. Longer periods of storage led to a gradual decrease in marrow viability measured in this way.

Since melphalan was undetectable in plasma after 2 h and in urine after 6 h and non-cryopreserved, heparinized marrow will grow control numbers of granulocytic colonies after 8 h storage at 4°C, 8 h was chosen as the

FIG. 4.—Granulocytic colony formation as a function of length of time marrow is stored at 4°C.

time to reinfuse the marrow after melphalan administration.

*Patients*.—Eight patients, details of whom are given in Table I, were treated with 140 $mg/m_2$ melphalan i.v. followed by a marrow autograft. All had widely disseminated malignant melanoma which was sufficiently advanced to require palliation but were otherwise in good physical condition, and all were under the age of 65. Patients were excluded from the study if they had impaired renal function, marrow infiltration, or if the bone scan or radiological skeletal survey showed bone involvement.

TABLE I.—*Clinical details of* 8 *patients with malignant melanoma treated with* 140 $mg/m^2$ *melphalan i.v. and marrow autograft*

| Case | Age | Sex | Site of primary | Length of history | Previous treatment | Actual dose of melphalan (mg) | Pre-treatment deposits | Response to treatment |
|---|---|---|---|---|---|---|---|---|
| 1 | 49 | M | Right foot | 3 years | Excision of primary | 250 | Regional lymph node distant cutaneous and lung | No regression. Died after 12 months |
| 2 | 55 | F | Left leg | 3 years | Left inguinal block dissection | 230 | Extensive cutaneous left leg | Minor (<50%) regression skin nodules for 2 months. Died after 8 months |
| 3 | 35 | F | Left arm | 17 months | Left axillary block dissection. Adjuvant DTIC+ Vincristine | 210 | Extensive intra-abdominal disease | Relief of pain with complete clearing of abdominal ultrasonogram for 3 months. Died after 5 months |
| 4 | 33 | M | Back | 16 months | Bilateral axillary block dissections. Adjuvant DTIC+ Vincristine. CCNU | 300 | Widespread cutaneous | Complete regression of nearly all skin deposits for 2 months |
| 5 | 29 | M | Left conjunctiva | 7 years | Excision of primary | 220 | Skin, lung and liver | Minor (<50%) regression of skin and lung deposits. Died after 3 weeks with extensive intra-abdominal disease and *E. coli* septicaemia |
| 6 | 34 | F | Neck | 18 months | Left cervical block dissection | 230 | Skin, lung, extensive liver | >50% regression of skin deposits but concurrently progressing liver disease |
| 7 | 36 | F | Back | 7 years | Excision of primary | 200 | Widespread skin plus lung | >50% regression of skin and lung deposits for 2 months |
| 8 | 35 | F | Right shoulder | 2 years | Right axillary block dissection | 240 | Massive recurrence right axilla. Multiple liver metastases | Complete regression of hepatic and locally recurrent disease for 4 months |

*Procedure.*—Under a light general anaesthetic a urinary catheter and central venous pressure line were introduced. The patients were heparinized with a single dose of 1,500 $u/m^2$ to facilitate marrow aspiration. They were placed supine and marrow and blood were aspirated from the anterior iliac crests through heparinized Salah marrow aspiration needles into 20 ml sterile heparinized syringes. After 200–300 ml of blood/marrow had been aspirated the patients were placed face down and the posterior iliac crests were sampled. Finally the sternum was used as a source of marrow. Between 2 and 4 ml of marrow was aspirated from each site. Multiple skin punctures were unnecessary, as the needles could be moved around under the skin, and usually only 2 skin punctures were needed on each side. Marrow was expressed from the syringes into a sterile plastic bag until $2\text{–}5\times10^8$/kg nucleated marrow cells had been harvested (200–500 ml of blood and marrow).

When the marrow harvest was complete the mixture of heparinized blood and marrow was placed in a 4°C refrigerator and the patient returned to the ward where 140 mg/$m^2$ melphalan was given i.v. The drug is supplied with its own diluent, and was made up immediately before injection, and it was injected as a bolus into the side arm of a fast running drip. (It can be very painful if a large vein is not used and the injection is not made slowly).

The melphalan was followed by 20 mg frusemide i.v. and 2 l of fluid were given i.v. over the next 3 h in order to prevent a high urinary melphalan concentration. Anti-emetics were given as required.

Hypotensive episodes occurred in the first 2 patients within 12 h of this procedure, probably due to a combination of the anaesthetic and the loss of blood aspirated with the marrow, and 2 units of blood were therefore given rapidly in the theatre to subsequent patients, whose pulse, blood pressure and central venous pressure were recorded every 15 min and urine output hourly until the marrow was returned. After the adoption of this routine, there were no further hypotensive episodes.

Eight hours after the melphalan had been given the blood and marrow were reinfused i.v. The marrow was unfiltered apart from its passage through the 300 μm filter in a standard blood-giving set. It was returned to the patient as fast as possible.

*Management of patients after treatment.*—Patients were barrier-nursed in single rooms during the period of neutropenia, and non-absorbed antibiotics were given orally to suppress potential gut pathogens (Framycetin, colistin and nystatin—Storring *et al.*, 1977). Febrile episodes were treated promptly with broad-spectrum i.v. antibiotics, and platelet concentrates were given when indicated.

### RESULTS (II)

### *Complications of treatment*

All patients were nauseated, and vomited within 4 h of treatment, and of the anti-emetics used 5 mg haloperidol i.v. appeared the most effective. Otherwise there were no immediate complications, apart from the hypotensive episodes mentioned above, and pain or discomfort from the marrow aspiration sites were not appreciable. After 5–6 days there was a marked loss of appetite, associated in most cases with nausea and depression, which persisted throughout the period of marrow hypoplasia. Alopecia developed during the 3rd week. More serious complications were febrile episodes, occurring in all patients during the 2nd week and from 2 of whom organisms were isolated in blood cultures (*Staph. aureus* and *E. coli* respectively) and transient elevation of blood urea in 2 patients (peak blood urea levels 18·0 and 24·4 mM).

Fever resolved when the granulocyte count rose above 400/$mm^3$, so that the ungrafted patients had longer periods of pyrexia than the grafted ones.

Both patients whose blood urea levels were raised after high-dose melphalan had high urinary concentrations of the drug during the first 2 h after its administration, and once the induction of a diuresis with frusemide and i.v. saline became routine practice there were no further cases.

### *The effect of treatment upon the tumour*

One patient (Case 8, Table I) achieved complete remission. She had a large axillary nodal recurrence with extension into breast tissue and multiple liver metastases. She remained in complete remission for 4 months after treatment, and then developed an axillary recurrence which was treated with radiotherapy. Seven months after treatment there was still no evidence of recurrence of the liver metastases. A second patient (Case 3) had disease limited to the retroperitoneal tissues and her abdominal ultrasonogram cleared completely after high-dose melphalan, although minimal disease persisted on lymphography. Of the remainder, 2 showed more than 50% regression of measurable skin deposits, as did a third whose liver metastases progressed nevertheless. One patient had no response at all, and the other 2 had transient regressions of skin deposits. None of these partial regressions was sustained for longer than 3 months.

FIG. 5.—Effect of marrow autotransplantation to 8 patients (- - - -) on platelet and leucocyte recovery after i.v. melphalan (140 mg/m²) compared with 4 patients receiving lower doses of melphalan (60–125 mg/m²) and no graft (——). Bars show ± s.d.

*The effect of treatment on blood count*

The mean granulocyte and platelet counts in the 8 grafted patients who received 140 mg/m² of melphalan are shown in Fig. 5, and the actual granulocyte

TABLE II.—*Granulocyte counts and drug dosage in grafted and ungrafted patients after i.v. melphalan on Day 0*

| Patient (mg/m² of melphalan) | Ungrafted patients No. of days from melphalan injection | | | | | | |
|---|---|---|---|---|---|---|---|
| | 0 | 6 | 10 | 16 | 22 | 26 | 32 |
| 1. (60) | 3000 | 2000 | 400 | 0 | 60 | 170 | 400 |
| 2. (100) | 4000 | 800 | 0 | 30 | 320 | 1000 | 3000 |
| 3. (100) | 5100 | 3700 | 0 | 0 | 40 | 560 | 3200 |
| 4. (125) | 12300 | 5000 | 300 | 0 | 200 | 570 | 3000 |
| Mean | 6100 | 2900 | 175 | 7 | 155 | 575 | 2400 |

| Patient | Grafted patients (all receiving 140 mg/m² melphalan) No. of days from melphalan injection | | | | | | |
|---|---|---|---|---|---|---|---|
| | 0 | 6 | 10 | 14 | 16 | 20 | 22 |
| 1. | 4600 | 1700 | 300 | 900 | 1700 | — | 2000 |
| 2. | 6500 | 350 | 60 | 460 | 1050 | | 2000 |
| 3. | 6700 | 1100 | 150 | 150 | 200 | 1050 | 1530 |
| 4. | 8000 | 300 | 150 | 820 | 2100 | — | — |
| 5. | 5000 | 1400 | 400 | 80 | 300 | 1000 | 1500 |
| 6. | 8360 | 700 | 200 | 300 | 700 | 950 | — |
| 7. | 8000 | 950 | 1400 | 1200 | 2500 | — | — |
| 8. | 3000 | 940 | 100 | 100 | 1500 | 1200 | 1200 |
| Mean | 6260 | 930 | 345 | 500 | 1250 | 1050 | 1650 |

counts and the dose of melphalan to each patient are shown in Table II. These are compared with those in the 4 patients receiving melphalan alone in doses ranging from 60 to 125 mg/$m^2$ i.v. Note that all the grafted patients received higher doses of melphalan than the ungrafted ones, and yet there was faster recovery of the granulocyte count in the grafted patients, an effect that can only be due to the marrow graft.

After melphalan treatment in both groups of patients, the total leucocyte count remained normal for the first 5 days, and then fell rapidly to a few hundred/$mm^3$. In both groups of patients the total granulocyte counts had fallen to less than 500/$mm^3$ by Day 10, with the exception of one grafted patient in whom the lowest recorded granulocyte count was 950/$mm^3$ on Day 6. By Day 16, 3/4 of the non-grafted patients had no detectable peripheral blood granulocytes, whereas of the grafted patients 2 had counts of more than 2000/$mm^3$, and 5 had counts of more than 1000/$mm^3$. By Day 20 all the grafted patients had granulocyte counts of 950/$mm^3$ or more. None of the non-grafted patients had a count of more than 400 at Day 22. The number of days taken to establish a granulocyte count of 800/$mm^3$ or greater was measured for each patient. The 2 groups were significantly different by the Mann–Whitney *U* test ($P=0{\cdot}01$). There was no significant acceleration of platelet recovery in the grafted patients. The reason for this is not known, but it is well known that in patients receiving marrow allografts from sibling donors a similar tardiness in platelet recovery is seen compared with the rate of granulocyte recovery. Possibly it is more difficult for megakaryocytic stem cells to become established and to mature when transplanted. However, this is less of a problem, since supportive treatment with platelet transfusions is readily available and easily given.

### DISCUSSION (II)

This study shows that autologous, unpreserved marrow infused 8 h after high-dose i.v. melphalan accelerates marrow recovery, and this is reflected most markedly in the recovery of peripheral-blood granulocytes, where the time taken for recovery to a mean count of 800/$mm^3$ in the grafted patient was 7 days, compared with 18 days in the non-grafted ones.

The effect upon the tumours was disappointing, apart from one patient in whom there was a complete remission. From this standpoint we have done little to answer an outstanding question in cancer chemotherapy—does increasing the dose of drugs increase the response of tumours? Experience with chemosensitive transplanted animal tumours has shown that this is so, but as Tattersall & Tobias (1976) have pointed out, there is little evidence in man that increasing the dose of most clinically useful antitumour drugs does more than increase toxicity to the patient. Clearly, in this context, the choice of both tumour and drug is likely to be important and, although we have found that malignant melanoma is relatively unresponsive to high-dose melphalan, other tumours more sensitive to conventional doses of alkylating agents, such as ovarian cancer, lymphomas, neuroblastoms and breast cancer, may prove to be more responsive.

The choice of drug for very-high-dose chemotherapy is also bound to be limited by the effect upon normal tissues other than the marrow, even if means are available to protect or rescue the marrow from drug damage. For example, it is unrealistic to think of increasing the dose of the many classes of drugs by more than about twice the upper limit of conventional dosage, since vinca alkaloids would produce prohibitive neurotoxicity, anthracycline antibiotics cardiotoxicity, nitrosoureas hepatoxicity and fluorouracil gastrointestinal toxicity. Even the well known exception, methotrexate, which can be given in enormous doses with folinic acid rescue, has not been shown convincingly to have an increased therapeutic effect when used in this way. In practice, we are left with alkylating agents and even some of these, such as cyclophosphamide and ifosphamide, produce urothelial toxicity which prohibits their use in very large doses.

Finally, melphalan itself could not be used in doses much greater than those employed in this study since damage to the gastrointestinal tract would supervene. Studies in sheep (Millar *et al.*, 1978) have shown that melphalan gut toxicity can be reduced by the use of small "priming" doses of cyclophosphamide given before the melphalan, and we have shown a similar sparing effect upon human marrow (Hedley *et al.*, 1978), but at present there is no evidence that this effect can be produced in human gut and, even if it could, it is not known by how much the melphalan dose could be increased nor whether such an increase would be therapeutically valuable.

Our future policy will be cautiously to explore the use of this treatment for tumours known to be more sensitive than malignant melanoma to alkylating agents and in whom resistance to conventionally employed agents has arisen.

Dr Hedley is supported by locally organized Clinical Research Funds.

Drs Gordon and Millar are supported by a programme grant from the Cancer Research Campaign and Medical Research Council.

Dr Milsted was a Gordon Hamilton Fairley Fellow.

### REFERENCES

ARIEL, I. M. & PACK, G. T. (1962) Treatment of disseminated melanoma with phenylalanine mustard and autogenous marrow transplants. *Surgery*, **51**, 583.

CLIFFORD, P., CLIFT, R. A. & DUFF, J. K. (1961) Nitrogen mustard therapy combined with autologous marrow infusion. *Lancet*, i, 687.

HEDLEY, D. W., McELWAIN, T. J., MILLAR, J. L. & GORDON, M. Y. (1978) Acceleration of bone marrow recovery by pre-treatment with cyclophosphamide in patients receiving high dose melphalan. *Lancet*, ii, 966.

HUMBLE, J. G., NEWTON, K. A., MELLOR, H. & PEGG, D. E. (1975) Prolonged survival in disseminated seminoma treated to aplasia by wide field x-radiotherapy and phenylalamine mustard, with autologous bone marrow cell transfusion cover. *Clin. Oncol.*, **1**, 235.

LUCE, J. K. (1975) Chemotherapy of melanoma. *Semin. Oncol.*, **2**, 179.

MILLAR, J. L., PHELPS, T. A., CARTER, R. L. & McELWAIN, T. J. (1978) Cyclophosphamide pretreatment reduces the toxic effect of high-dose melphalan on the intestinal epithelium in sheep. *Eur. J. Cancer*, **11**, 1283.

PIKE, B. L. & ROBINSON, W. A. (1970) Human bone marrow colony growth in agar gel. *J. Cell Physiol.*, **76**, 77.

TATTERSALL, M. H. N., JARMAN, M., NEWLANDS, E. S., HOLYHEAD, L., MILSTED, R. A. V. & WEINBERG, A. (1978) Pharmacokinetics of melphalan following oral or intravenous administration in patients with malignant disease. *Eur. J. Cancer*, **14**, 507.

TATTERSALL, M. H. N. & TOBIAS, J. S. (1976) How strong is the case for intensive cancer chemotherapy? *Lancet*, ii, 1071.

TOBIAS, J. S., WEINER, R. S., GRIFFITHS, C. T., RICHMAN, C. M., PARKER, L. M. & YANKEE, R. A. (1977) Cryopreserved autologous marrow infusion following high dose cancer chemotherapy. *Eur. J. Cancer*, **13**, 269.

WEAVER, P. C., WRIGHT, J., BRANDER, W. L. & WESTBURY, G. (1975) Salvage procedures for locally advanced malignant melanoma of the lower limb (with special reference to the role of isolated limb perfusion and radical lymphadenectomy). *Clin. Oncol.*, **1**, 45.

WESTBURY, G., HUMBLE, J. G., NEWTON, K. A., SKINNER, M. E. G & PEGG, D. E. (1959) Disseminated malignant melanoma. Response to treatment by massive dosage of a cytotoxic agent combined with autogenous marrow replacement. *Lancet*, i, 968.

ZIEGLER, J. L., DEISSEROTH, A. B., APPLEBAUM, F. R. & GRAW, R. G. (1977) Burkitt's lymphoma —a model for intensive chemotherapy. *Semin. Oncol.*, **4**, 317.